# Single locking compression plate fixation of extra-articular distal humeral fractures

**DOI:** 10.1007/s10195-014-0325-8

**Published:** 2014-10-19

**Authors:** Malhar N. Kumar, M. R. Ravishankar, Ravikiran Manur

**Affiliations:** HOSMAT Hospital, 45, McGrath Road, Bangalore, 560025 India

**Keywords:** Distal humerus fractures, Locking compression plate, Plate osteosynthesis of humerus, Metaphyseal fractures of humerus

## Abstract

**Background:**

Earlier literature on fixation of distal third humeral fractures describes the use of elaborate modification of existing implants, custom-made implants and dual plating. These modifications have the disadvantages of limitations of hardware availability and cost as well as longer surgical exposure to accommodate the plates. The aim of this study was to assess the effectiveness of osteosynthesis of extra-articular diaphyseal fractures of the distal third of the humerus using a single 4.5-mm locking compression plate (LCP) with two-screw purchase in the distal fragment.

**Materials and methods:**

We performed internal fixation of distal third extra-articular humeral fractures in 22 adult patients using 2–3 lag screws neutralized with a single 4.5-mm locking compression plate with only two screws in the distal fragment. The mean follow-up period was approximately 1.6 years.

**Results:**

Fractures united in all 22 patients with minimal complications. The mean time to union of fracture was 13 weeks. The Mayo elbow score and the DASH scores were in the excellent and good category in all patients at final follow-up.

**Conclusions:**

Our study showed that it is possible to obtain excellent outcomes in distal third fractures using only a single 4.5-mm LCP with two-screw (4-cortices) purchase in the distal fragment. The disadvantages inherent in the previous methods can be avoided with the use of the present technique. This technique obviates the need for the use of customized distal humeral implants and modified implants in most patients.

**Level of evidence:**

Level IV.

## Introduction

Fractures of the distal third of the humerus are challenging injuries due to their peri-articular location, small size of the distal bone fragments, and the osteopenic quality of the bone in older adults. Methods of management of distal humerus fractures include conservative management using plaster cast immobilization or functional bracing, plate osteosynthesis and intra-medullary nailing [1–4]. Stewart et al. proposed that fractures of the distal-third humerus shaft should not be treated by hanging cast because angulation is difficult to control [1]. Sarmiento et al. treated 85 extra-articular comminuted distal-third humeral fractures with a functional brace. The nonunion rate in their series was 4 % and the malunion rate was 16 % (varus angulation in the majority). A decrease in the range of motion at the elbow and shoulder was another significant problem in their series [2]. Jawa et al. compared the use of functional bracing and plate fixation for extra-articular distal-third diaphyseal fractures of the humerus. They concluded that for extra-articular distal-third diaphyseal humeral fractures, surgical treatment achieves more predictable alignment and potentially quicker return of function but risks iatrogenic nerve injury and infection and the need for reoperation [3].

It is difficult to manage extra-articular distal humerus fractures with locking intra-medullary nails. The flat cross section of the distal humerus with a narrow medullary canal makes it difficult to insert intra-medullary nails and increases liability for comminution of the distal fragment during nail insertion. The short distal fragment makes it difficult to achieve stable fixation with distal interlocking. Radial nerve injury, if present, cannot be addressed without a separate incision. Plate osteosynthesis has distinct advantages in the distal humerus, and compression plating has been established as a successful modality for the surgical treatment of humerus fractures [4].

Recommendations for improving stability of plate fixation include plate thickness of >3.5 mm (a large-fragment plate) for most adults, and at least four screw holes in both the proximal and distal fragments [5]. However, adhering to these principles becomes difficult in distal humeral shaft fractures, especially those around the metaphyseal transition zone between the shaft and the supracondylar ridges. Fixation with three or four screws in the distal fragment is difficult as longer plates tend to impinge on the olecranon fossa.

Livani et al. reported the use of percutaneous osteosynthesis in a small series of six patients with distal humerus fractures with preoperative radial nerve palsy [6]. They used a dynamic compression plate for fixation (with two screws on either side of the fracture). The purpose of our study was to assess the effectiveness of a contoured standard 4.5-mm locking compression plate (LCP), with the use of only two screws in the distal fragment, in the management of distal-third fractures of the humerus. Inter-fragmentary screws were used wherever possible and the plate was used as a neutralization plate. If this method is effective in achieving fracture union with minimal rates of complications, it offers the advantages of the use of a standard and easily available implant, avoiding fixation beyond the olecranon fossa and avoiding extension of the incision beyond the elbow crease.

## Materials and methods

A prospective study was conducted between October 2011 and December 2012. Permission was obtained from the hospital ethics committee prior to commencing the study. Informed written consent was obtained from the patients prior to the study. The patient cohort consisted of 22 patients with distal-third diaphyseal humerus fractures. All adult patients with closed extra-articular fractures of the distal third of the humerus were included in this study. Patients with open fractures, pathological fractures, fractures with articular or intercondylar extension, floating elbow injury and children with distal humerus fractures were excluded from the study. The mean age of the patients was 32.6 years (21–58 years); almost 60 % of the patients were aged 21–30 years. 14 fractures were on the left side and 8 on the right side. The predominant mode of injury was road traffic accident (17 patients). In three patients, the fracture was the result of a fall and in two patients, the fracture was due to assault. All patients were operated on within 48 h of injury. The fractures were classified based on the anteroposterior and lateral radiographs. The OTA classification is shown in Table 1. An LCP was chosen (even though the bone quality of our patients was good due to the younger age) to ensure reliable fixation with only two screws distally.

**Table I:** Orthopaedic Trauma Association (OTA) classification of fractures

OTA fracture subtype	No. of patients	Percentage
12A1.3	5	22.7
12A2.3	2	9.1
12B1.3	7	31.8
12B2.3	5	22.7
12B3.3	1	4.5
12C1.3	2	9.1
Total	22	100

**Fig. 1:**
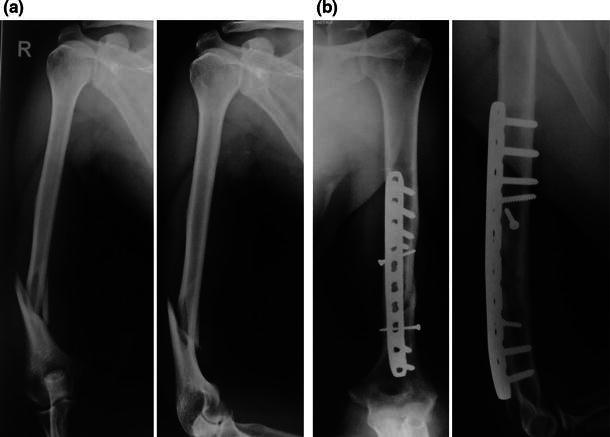
**a** Anteroposterior and lateral views showing an oblique fracture with medial butterfly fragment. **b** Immediate post-operative radiographs showing fixation with two lag screws and pre-contoured LCP with two locking screws in the distal fragment

**Fig. 2:**
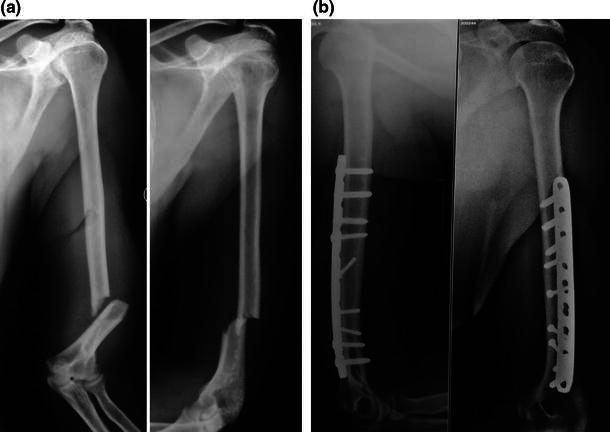
**a** Anteroposterior and lateral views showing a transverse fracture of the distal third of the humerus. **b** Post-operative anteroposterior and lateral radiographs showing sound union at 5 months following fixation with LCP

**Fig. 3:**
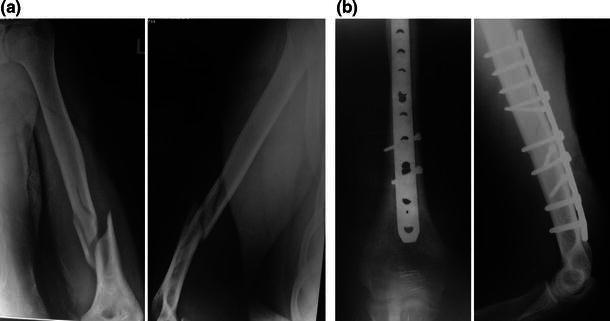
**a** Anteroposterior and lateral views showing a distal-third fracture with medial comminution and proximal extension of the fracture line. **b** Post-operative anteroposterior and lateral views showing complete union at 4.5 months; only two locking screws are in the distal fragment

All patients were treated with LCP fixation with a posterior midline triceps-splitting approach. The patient was positioned in the lateral decubitus position with the elbow flexed over a well-padded radiolucent bolster. The incision stopped short of the tip of the olecranon. The triceps was split in the midline until the apex of the olecranon fossa, and no dissection was performed distally. The triceps was not reflected from the medial or lateral supracondylar ridges. The radial nerve was dissected in the region of the spiral groove, traced until the junction of the middle and distal thirds of the humerus. The fracture was stabilized using two or three 3.5-mm lag screws, and a 4.5-mm LCP was used as a neutralization plate (Figs. 1, 2, 3). Two lag screws were used in 14 patients with short oblique fracture patterns. Three lag screws were used in the remaining eight patients with a long oblique/spiral fracture pattern. The plate was contoured intra-operatively to match the dorsal surface of the humerus accurately. Bending was performed at the site of the dynamic compression hole. Following plate fixation, the radial nerve was repositioned superficial to the plate and the wound was closed in layers. A long arm slab was applied for 3 weeks following the operation for pain relief. Physiotherapy including active assisted range of motion exercises was started 1 week post-operatively. Between 1 and 3 weeks following the operation, an active and gentle passive range of motion exercises was performed twice a week under the direct supervision of the surgeon (the slab was removed and reapplied). From the fourth week onwards, patients were allowed to perform active and gentle passive exercises on their own with the aid of the physiotherapist. Lifting of light weights was permitted only after complete radiological union was seen at the fracture site.

Patients were followed up clinically and radiologically every 6 weeks until fracture union. Functional outcome was measured by the ‘Mayo Elbow Performance Index’ (MEPI) and the ‘Disabilities of Arm, Shoulder and Hand’ (DASH) questionnaire at final follow-up. The MEPI is one of the most commonly used physician-based elbow rating systems [7]. This index consists of four parts—pain (with a maximum score of 45 points), ulnohumeral motion (20 points), stability (10 points) and the ability to perform five functional tasks (25 points). The DASH questionnaire is a standardized questionnaire which evaluates impairments and activity limitations, as well as participation restrictions for both leisure activities and work [8]. It includes questions about symptoms and disabilities of upper limb (30 items). Statistical analysis was performed using SPSS 20.0 software [version 17 Chicago, IL: SPSS, Inc.; 2008].

## Results

The mean duration of surgery was 110 ± 15.3 min (90–150 min). Average blood loss was 155 ± 25.5 ml (130–240 ml), measured using the surgical swab weighing technique. The mean duration of follow-up was 15.3 ± 1.3 months (14–17 months), with a minimum follow-up period of 14 months. Radiological union was evident by an average of 13.5 ± 1.46 weeks (10–17 weeks). Complications were found in 2 of the 22 patients—one patient had signs of early myositis ossificans and another patient had a broken lag screw (the fracture had clinically and radiologically united). There were three patients with pre-operative radial nerve palsy, diagnosed in the emergency department; operative findings showed radial nerve contusion in all the three patients. There were no post-operative/iatrogenic radial nerve palsies. The three patients with pre-operative radial nerve palsy recovered within a mean period of 5 months. All patients had full range of shoulder and elbow motion, except one patient who had loss of extension of 10 degrees in the elbow.16 out of 22 patients (72.7 %) in our series had excellent scores and six (27.3 %) had good scores on the MEPI scoring system. The mean DASH score in our series was 14.3 (SD ± 8.3). The DASH score was <15 in 16 out of 22 patients (5.0–14.20) and between 15 and 30 in six patients (15.80–29.20).

## Discussion

At present, there is a paucity of literature on the management of distal-third diaphyseal fractures of the humerus. The current study deals with lower metaphyseal fractures of the humerus (extra-articular distal humerus fractures) treated using a 4.5-mm LCP (contoured intra-operatively) as a neutralization plate with 3.5-mm lag screws, using the posterior triceps-splitting approach. Various modifications of plate osteosynthesis have been introduced. These include the use of a modified lateral tibial head buttress plate, custom-made ‘hybrid’ locking plates, double reconstruction plates and anterior plating of the distal humerus [9–11]. Each of these methods has its own disadvantages both in surgical techniques as well as in the choice of implants. Levy et al. [9] modified the Synthes^®^ Lateral Tibial Head Buttress Plate for use at the distal humerus. An ipsilateral Lateral Tibial Head Buttress Plate was modified using a high-speed rotary diamond-cutting tool to remove the posterior hole of the proximal expanded section of the plate. The resulting sharp edges were rounded off with a diamond-cutting wheel. The plate was then bent so that the bend in the proximal section of the plate was reversed. This resulted in a 4.5-mm limited contact dynamic compression plate (LC-DCP) with a distal angular offset of approximately 22° that allowed the modified plate to be placed on the lateral column of the distal humerus. The authors reported good results in their series. The problem with this approach is the necessity for elaborate modification of an existing design or the necessity for bulk production of such a modified design.

Spitzer et al. [10] used a custom-made ‘hybrid’ locking plate for difficult fractures of the meta-diaphyseal humeral shaft. This was a special plate prepared for use by the author with 4.5-mm locking holes at one end and a cluster of 3.5-mm locking holes at the other end (distal).The outcome was excellent in their series; however, this approach also involves modification of existing designs and their bulk production for universal use. Zhiquan et al. [11] treated 13 distal third humeral shaft fractures with minimally invasive percutaneous osteosynthesis (MIPO). Fractures were reduced by closed means and fixed with a long narrow 4.5-mm dynamic compression plate introduced through two small incisions away from the fracture site. The plate was fixed on the anterior aspect of the humerus under fluoroscopy guidance. The radial nerve was not exposed during this procedure. They reported that the fractures united with a mean healing time of 16.2 weeks, a little longer than the reported time of 9–12 weeks in posterior open plating of the humerus. Disadvantages of this approach are that the radial nerve is not visualized directly during the exposure and, biomechanically, the posterior surface of the humerus is considered better for plate application especially of distal-third fractures. Schatzker and Tile listed four reasons for plating the distal humerus posteriorly—the posterior surface of the distal humerus provides a flat surface suitable for plating; placement of the most distal screws from a posterior approach allows direct visualization and avoids the antecubital fossa; posterior placement allows for the plate to extend distally permitting additional screw placement; and the posterior approach provides the option of double plating [12]. Livani et al. [6] obtained good results following minimally invasive percutaneous DCP fixation of distal humerus fractures in six patients with radial nerve palsy. We chose an LCP due to the presence of significant comminution in many of our patients. The open surgical approach that we used required more soft tissue stripping which made stable fixation mandatory. Since the majority of our patients had no radial palsy pre-operatively, nerve exploration and protection required an open approach.

Prasarn et al. [13] treated extra-articular fractures of the distal third of the humerus with dual plates from a single posterior midline incision (2.7- and 3.5-mm pelvic reconstruction plates). The average time to union was 11.5 weeks and the mean elbow flexion/extension arc was 4°–131°. Possible disadvantages of this approach are the necessity to reflect the triceps to accommodate plate application on the lateral column, and the need for using two plates to secure reduction. The 2.7- and 3.5-mm plates used in this series tend to be less strong than 4.5-mm compression plates.

Advantages of our technique are that fracture stabilization is achieved with a single 4.5-mm LCP without any modification of the implant except for slight contouring. The posterior approach dissection was limited up to the olecranon fossa hence avoiding triceps fibrosis/elbow stiffness as it was not necessary to expose the lateral column until the distal tip. Use of a 4.5-mm LCP obviates the need for double plating and simplifies the procedure. Contouring allows the plate to match the posterior surface of the humerus and prevents the tip of the plate from rising above the humerus just proximal to the olecranon fossa. Secondly, it minimizes stress on the skin and soft tissues overlying the plate [14]. Bending was performed at the level of the dynamic hole in the plate as recommended by Smith et al. [15]. Since the distal fixation relies on only two screws, quality of bone is important and the technique is best avoided in elderly patients with poor bone quality and in highly comminuted fractures. Our results were excellent in terms of fracture union as well as elbow and shoulder range of motion. We had two complications, namely breakage of a lag screw in one patient and early myositis ossificans in the second patient; however, the patients were not seriously affected and the quality of the results did not suffer due to these complications. Our post-operative protocol consisted of immobilization of the elbow in a long arm slab for 3 weeks. However, the slab was removed every week and the elbow was mobilized under the direct supervision of the surgeon. This subsequently proved to be helpful in the early recovery of range of movement. There is no need for elaborate modification of existing implants and no need for the use of custom-made implants. It can be argued that use of only two screws in the distal fragment might compromise the stability of fixation. It has been shown by Hak et al. [16] that two locking screws per segment are sufficient and the addition of a third screw in the locked plate construct did not add to the mechanical stability in axial loading, bending, or torsion. It is possible to insert at least two locking screws in the distal fragment in the vast majority of distal humeral fractures.

We conclude that the use of one or two lag screws along with a single posteriorly placed 4.5-mm contoured locking compression plate having at least two locking screws in the distal fragment provides sufficient rigid fixation in distal metaphyseal fractures of the humerus. The dissection does not extend beyond the apex of the olecranon fossa. The implant stops well short of the olecranon fossa. Excellent results can be achieved in these fractures without the use of dual plating and without the need for expensive customized implants or elaborately modified implants. Careful patient selection is important for this technique and indiscriminate use of single-plate fixation should be avoided. Physiologically, young patients with good bone quality and good motivation for post-operative physiotherapy are suitable for this technique. Patients with open fractures, highly comminuted fractures, fractures with intercondylar extensions and pathological fractures are not suitable for this type of fixation.
